# Inhibition of CPT2 exacerbates cardiac dysfunction and inflammation in experimental endotoxaemia

**DOI:** 10.1111/jcmm.15809

**Published:** 2020-09-07

**Authors:** Marina Makrecka‐Kuka, Stanislava Korzh, Melita Videja, Reinis Vilskersts, Eduards Sevostjanovs, Olga Zharkova‐Malkova, Pavel Arsenyan, Janis Kuka, Maija Dambrova, Edgars Liepinsh

**Affiliations:** ^1^ Latvian Institute of Organic Synthesis Riga Latvia; ^2^ Faculty of Pharmacy Riga Stradins University Riga Latvia

**Keywords:** acylcarnitine, endotoxaemia, fatty acid oxidation, heart, mitochondria

## Abstract

The suppression of energy metabolism is one of cornerstones of cardiac dysfunction in sepsis/endotoxaemia. To investigate the role of fatty acid oxidation (FAO) in the progression of inflammation‐induced cardiac dysfunction, we compared the effects of FAO‐targeting compounds on mitochondrial and cardiac function in an experimental model of lipopolysaccharide (LPS)‐induced endotoxaemia. In LPS‐treated mice, endotoxaemia‐induced inflammation significantly decreased cardiac FAO and increased pyruvate metabolism, while cardiac mechanical function was decreased. AMP‐activated protein kinase activation by A769662 improved mitochondrial FAO without affecting cardiac function and inflammation‐related gene expression during endotoxaemia. Fatty acid synthase inhibition by C75 restored both cardiac and mitochondrial FAO; however, no effects on inflammation‐related gene expression and cardiac function were observed. In addition, the inhibition of carnitine palmitoyltransferase 2 (CPT2)‐dependent FAO by aminocarnitine resulted in the accumulation of FAO intermediates, long‐chain acylcarnitines, in the heart. As a result, cardiac pyruvate metabolism was inhibited, which further exacerbated inflammation‐induced cardiac dysfunction. In conclusion, although inhibition of CPT2‐dependent FAO is detrimental to cardiac function during endotoxaemia, present findings show that the restoration of cardiac FAO alone is not sufficient to recover cardiac function. Rescue of cardiac FAO should be combined with anti‐inflammatory therapy to ameliorate cardiac dysfunction in endotoxaemia.

## 1 INTRODUCTION

Despite efforts to develop effective treatment strategies, sepsis accounts for approximately 20% of deaths worldwide.^1^ In 2017, approximately 50 million cases of sepsis were recorded globally, and 11 million sepsis‐related deaths were reported.^1^ The progression of cardiac dysfunction is an important component of multiple organ dysfunction syndrome, which is caused by sepsis and endotoxaemia. It has been shown that 13% of patients who are hospitalized due to sepsis experience at least one cardiovascular event and have a 30% higher risk of death.^2^ The mechanisms that contribute to cardiac dysfunction include increased inflammatory signalling and/or suppressed energy metabolism (both glucose and fatty acid).^3^ It has been shown that circulating inflammatory cytokines and inflammatory pathway activation may lead to impaired cardiac function.^4^, ^5^ However, attempts to improve cardiac function by targeting inflammatory pathways are not sufficient to decrease the mortality rate.^6^, ^7^, ^8^ Meanwhile, targeting energy metabolism could be an effective add‐on therapy for improving cardiac function during sepsis and endotoxaemia in the clinic.

Due to the high energy demand, the function of the heart is highly dependent on energy metabolism and, in particular, on mitochondrial oxidative phosphorylation. Although the heart can utilize different substrates, fatty acid oxidation contributes 60%‐90% of the ATP produced.^9^ It is well known that disturbances in fatty acid metabolism are strongly associated with impaired cardiac function; decreased fatty acid metabolism has been found in both animal and human studies of heart failure,^10^, ^11^, ^12^, ^13^ while patients with inborn disorders of fatty acid oxidation (FAO) suffer from cardiomyopathy.^14^ Inflammation, endotoxaemia and sepsis‐induced cardiac dysfunction are also associated with impaired FAO.^15^, ^16^, ^17^, ^18^ Overall, one might assume that the preservation of FAO during sepsis or endotoxaemia could be beneficial for heart function.

Studies demonstrating the beneficial effects of stimulated fatty acid metabolism on cardiac function during sepsis or endotoxaemia are mostly based on the use of knockout models and do not investigate the cardioprotective effects of pharmacological agents.^19^, ^20^, ^21^, ^22^ Thus, to date, there are very few studies demonstrating the effects of pharmacological stimulation of fatty acid utilization on both cardiac energy metabolism and cardiac function during sepsis or endotoxaemia. In the present study, several pharmacological agents were selected to investigate their effects on cardiac function and fatty acid metabolism in an experimental model of endotoxaemia. First, C75, an inhibitor of fatty acid synthase (FASN), has been shown to stimulate fatty acid utilization by increasing the activity of carnitine palmitoyltransferase 1 (CPT1; the rate‐limiting enzyme in mitochondrial FAO) and to ameliorate liver injury in the caecal ligation and puncture models of sepsis ^21^, ^23^; however, the effects of C75 on the heart had never been investigated. Second, the pharmacological activation of AMP‐activated protein kinase (AMPK, the modulator of energy metabolism) has been shown to stimulate fatty acid oxidation,^24^ although the effects of AMPK activation on cardiac function in an lipopolysaccharide (LPS)‐induced endotoxaemia model are controversial.^25^, ^26^ In addition, in the present study, an inhibitor of CPT2, aminocarnitine, was used to investigate the significance of limited mitochondrial FAO on cardiac function in the inflamed heart. It should be noted that in the present study, the compounds were administered 2 hours after LPS injection to ensure better translation of the results to the clinical settings.

## 2 MATERIALS AND METHODS

### 2.1 Animals and treatments

Fifty‐five female HsdWin:NMRI mice (8‐10 weeks old, 29‐30 g) were obtained from Envigo and adapted to local conditions for 2 weeks prior to the experiments. All the animals were housed under standard conditions (21‐23°C, 12‐hour light/dark cycle, 45%‐65% relative humidity) with unlimited access to food (R70 diet; Lactamin AB) and water. The experimental procedures were performed in accordance with the guidelines of the European Community as well as local laws and policies, and the procedures were approved by the Latvian Animal Protection Ethical Committee of the Food and Veterinary Service, Riga, Latvia. All studies involving animals were reported in accordance with the ARRIVE guidelines.^27^, ^28^ Data from previous experiments where LPS‐induced inflammation was determined were subjected to statistical power analysis and calculations indicated that depending on fatty acid metabolism assay n = 5 or 6 would produce significant result with power >0.95.

The animals were randomly separated into five experimental groups (n = 11): the control group, LPS‐induced endotoxaemia group, LPS + C75 (FASN inhibitor) group, LPS + A769662 (AMPK activator) group and LPS + Aminocarnitine (CPT2 inhibitor) group. A single injection of LPS from *Escherichia coli* 055:B5 (10 mg/kg, i.p.; Sigma‐Aldrich L4005 Lot#033M4054V) was used to induce endotoxaemia. The control animals received an injection of saline. Because the administration of LPS is known to reduce food intake, the animals were deprived of food from the time of LPS or saline injection until the end of the experiment to ensure equal conditions for the control and LPS‐treated animals. The access to water remained unlimited during the experiment. At 2 hours after LPS administration, C75 (5 mg/kg), A769662 (10 mg/kg), aminocarnitine (10 mg/kg) or vehicle (1% DMSO in 10% 2‐hydroxypropyl‐beta‐cyclodextrin in saline) was injected intraperitoneally. The doses of C75 and A769662 were chosen based on data from previously published studies,^21^, ^26^ while the dose of aminocarnitine was chosen based on our previous experimental data on the accumulation of long‐chain acylcarnitines in tissues after a single injection of the compound (unpublished data). C75 and A769662 were purchased from Tocris Bioscience and Carbosynth Ltd., respectively. Aminocarnitine was synthesized from l‐carnitine by a modified method (see Appendix S1) as described previously.^29^ Four hours after LPS administration, the animals (n = 6 per group) were used for echocardiography and in vivo palmitate metabolism studies or for mitochondrial function measurements (n = 5 per group). Animals were killed by decapitation, and both trunk blood and heart tissue samples were collected. Heart and plasma samples were collected and immediately used to assess mitochondrial function or were stored at −80°C until analysis. In addition, the body temperature was monitored and the blood glucose concentration was measured using an Accu‐Chek Instant blood glucose meter and strips (Roche Diagnostics).

### 2.2 Biochemistry

To obtain plasma, the blood samples were centrifuged at 1000 *g* at 4°C for 10 minutes and then stored at −80°C until analysis. The levels of interleukin 6 (IL‐6) and tumour necrosis factor α (TNFα) in the plasma were measured using ELISA kits from Invitrogen by Thermo Fisher Scientific and R&D Systems.

### 2.3 Isolation of RNA and quantitative RT‐PCR analysis

The total RNA from the cardiac tissues was isolated using TRI reagent (Sigma) according to the manufacturer's recommended protocol. First‐strand cDNA synthesis was performed using the High‐Capacity cDNA Reverse Transcription Kit (Applied Biosystems™) following the manufacturer's instructions. Quantitative RT‐PCR analysis of genes was performed by mixing the SYBR^®^ Green Master Mix (Applied Biosystems™), synthesized cDNA, and forward and reverse primers specific for IL‐6, IL‐1β, TNFα and β‐actin and running the reactions in an Applied Biosystems Prism 7500 instrument according to the manufacturer's recommended protocol. The relative expression levels of each of the genes of interest were calculated with the ∆∆Ct method and were normalized to the expression of the β‐actin gene. The primer sequences used for the quantitative RT‐PCR analysis are listed in Appendix S1.

### 2.4 Echocardiographic assessment

The mice were anaesthetized using 5% isoflurane dissolved in a mixture of oxygen and nitrous oxide (50/50 v/v). After the onset of anaesthesia, the concentration of isoflurane was decreased to 2.5%, the experimental animals were placed in a decubitus position, and the chest was shaved. M‐mode tracings of the left ventricle were recorded at the papillary muscle level using an iE33 ultrasonograph equipped with a linear L15‐7io transducer (Philips Healthcare).

### 2.5 Measurement of the levels of acylcarnitines

The cardiac acylcarnitine content was measured by ultra‐performance liquid chromatography MS/MS using the previously described method.^30^

### 2.6 Measurements of palmitate oxidation in vivo

To determine palmitate oxidation in vivo, 1 µCi of [9,10‐^3^H] palmitate (specific activity, 60 Ci/mmol) per mouse was administered subcutaneously. After 10 minutes, the mice were killed by cervical dislocation, and heart tissue homogenates (1:5 w/v in Milli‐Q water) were prepared. The samples were treated as previously described.^31^

### 2.7 Mitochondrial respiration measurements

The mitochondrial function was assessed in permeabilized cardiac fibres that were prepared as previously described.^15^ The mitochondrial respiration measurements were performed at 37°C using an Oxygraph‐2k (O2k; Oroboros Instruments) in MiR05 media (110 mmol/L sucrose, 60 mmol/L K‐lactobionate, 0.5 mmol/L EGTA, 3 mmol/L MgCl_2_, 20 mmol/L taurine, 10 mmol/L KH_2_PO_4_, 20 mmol/L HEPES, pH 7.1, 0.1% BSA essentially free of fatty acids).

Palmitoylcarnitine and malate (10 µmol/L and 0.5 mmol/L, respectively) were added to measure the FAO‐dependent mitochondrial respiration (FADH_2_(NADH)‐pathway; F(N)‐pathway) at LEAK (L), substrate‐dependent, state. Then, ADP was added to a concentration of 5 mmol/L to initiate oxidative phosphorylation‐dependent respiration (OXPHOS state). Next, pyruvate (5 mmol/L, complex I substrate, N‐pathway) was added to reconstitute FN‐pathway‐linked respiration and to measure pyruvate metabolism‐supported respiration in the presence of FAO substrate. Succinate (10 mmol/L, complex II substrate, S‐pathway) was added to reconstitute convergent FNS‐linked respiration, thus determining respiration rate at full (involving FADH_2_‐, NADH‐ and succinate‐linked pathways) electron transfer system OXPHOS capacity. Then, rotenone (0.5 µmol/L, inhibitor of complex I) and antimycin A (2.5 µmol/L, inhibitor of complex III) were added to determine the S‐linked respiration and residual oxygen consumption (ROX), respectively.

To determine the contribution of each substrate to the respiration rate, the flux control factor was calculated as follows:

$$1‐\frac{\text{Resp}.\text{rate}\;\;\text{before}\;\text{the}\;\text{addition}\;\text{of}\;\text{substrate}}{\text{Resp}.\text{rate}\;\;\text{after}\;\text{the}\;\text{addition}\;\text{of}\;\text{substrate}}.$$

### 2.8 Data analysis

All the data are expressed as the means ± SEM. Since n per group is lower than 7, Shapiro‐Wilk normality test was used. Afterwards, saline control and LPS control groups were compared using Student's *t* test to evaluate the effects of endotoxaemia model per se. Then, statistically significant differences in the mean values of LPS‐treated groups were evaluated using one‐way ANOVA followed by Dunnett's post‐test. *P* values <.05 were considered to indicate statistical significance. The statistical calculations were performed using GraphPad Prism software.

## 3 RESULTS

### 3.1 Endotoxaemia‐induced inflammation

The LPS‐treated mice developed clinical signs of endotoxaemia (Table 1). Compared with that of the control group, the body temperature of the LPS group was significantly decreased by 2.1°C at 4 hours after LPS administration. The blood glucose concentration was decreased by 2.8 mmol/L after LPS administration. Treatment with C75 and A769662 did not induce any changes in body temperature and blood glucose concentration. Meanwhile, administration of the CPT2 inhibitor aminocarnitine resulted in an additional significant decrease in body temperature and blood glucose compared to administration of LPS alone.

**TABLE 1:** Changes in body temperature and blood glucose concentration 4 h after LPS administration

	Group	Body temperature, °C	Blood glucose, mmol/L
Control	Saline	37.1 ± 0.8	6.2 ± 1.1
LPS	Control	35.0 ± 0.8*	3.4 ± 0.4*
	C75	34.8 ± 1.1	3.3 ± 0.7
	A769662	34.9 ± 0.9	3.2 ± 0.6
	Aminocarnitine	32.2 ± 0.7**	2.0 ± 0.9**

Note

Each value represents the mean ± SEM of 10 (saline control) or 11 animals.

* Significant difference between saline control and LPS control groups (Student's *t* test, *P* < .05).

** Significantly different from the LPS control group (ANOVA followed by Dunnett's test, *P* < .05).

**FIGURE 1:**
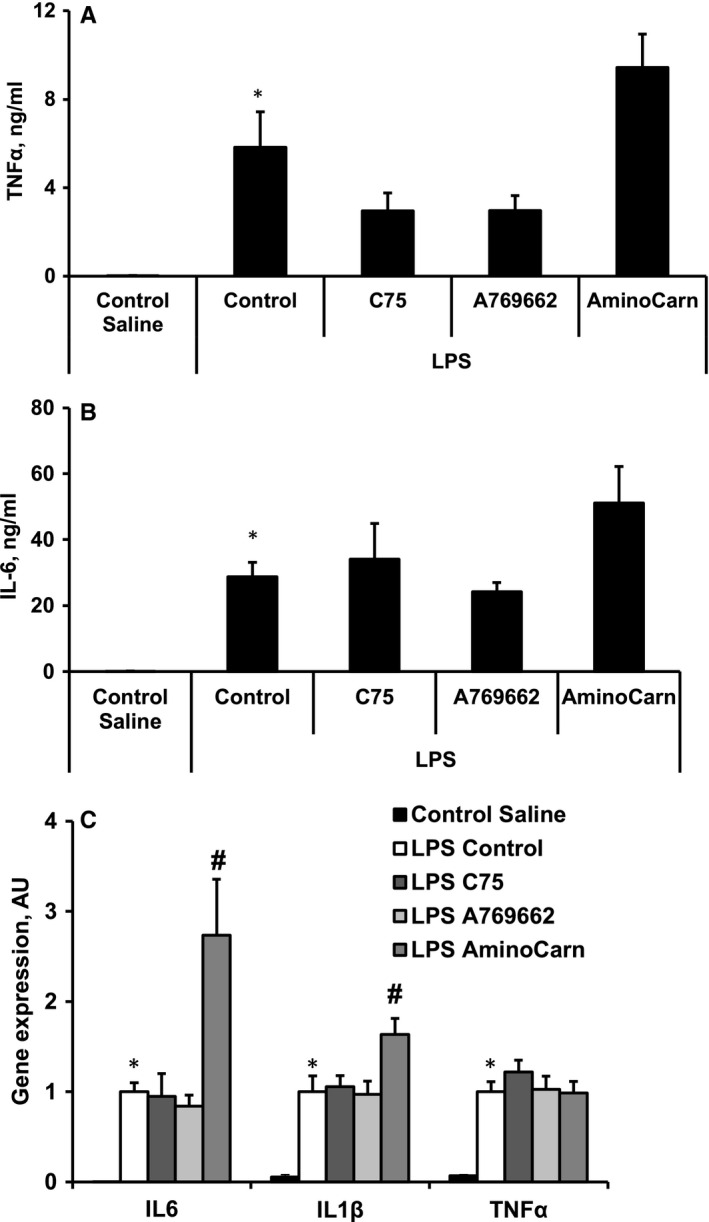
LPS‐induced increase in plasma markers, TNFα (A) and IL‐6 (B), and expression of genes related to inflammation in heart tissues (C). The treatment with aminocarnitine increased the expression of inflammation marker genes in the cardiac tissue (C). Each value represents the mean ± SEM of five animals for the plasma markers and 4‐5 animals for the gene expression analysis. *Significant difference between saline control and LPS control groups (Student's *t* test, *P* < .05). ^#^Significantly different from the LPS control group (ANOVA followed by Dunnett's test, *P* < .05)

To evaluate the severity of inflammation, the concentrations of inflammation markers in the plasma and the expression of inflammation‐related genes in the cardiac tissue were determined. After LPS administration, the concentrations of the inflammation markers TNFα and IL‐6 in the plasma were notably increased up to 6 and 29 ng/mL, respectively (Figure 1A,B). Measurement of gene expression (Figure 1C) demonstrated that after the administration of LPS, inflammation marker genes (IL‐6, IL‐1β and TNFα) were up‐regulated in the cardiac tissues. Treatment with C75 and A769662 tended to decrease the concentration of TNFα in the plasma by 49% (Figure 1A) but did not affect the expression of inflammation marker genes in the cardiac tissues (Figure 1C). In contrast, in the aminocarnitine‐treated group, the expression of inflammation marker genes in the cardiac tissues was significantly higher than those in the LPS group (Figure 1C).

### 3.2 Cardiac functionality

**FIGURE 2:**
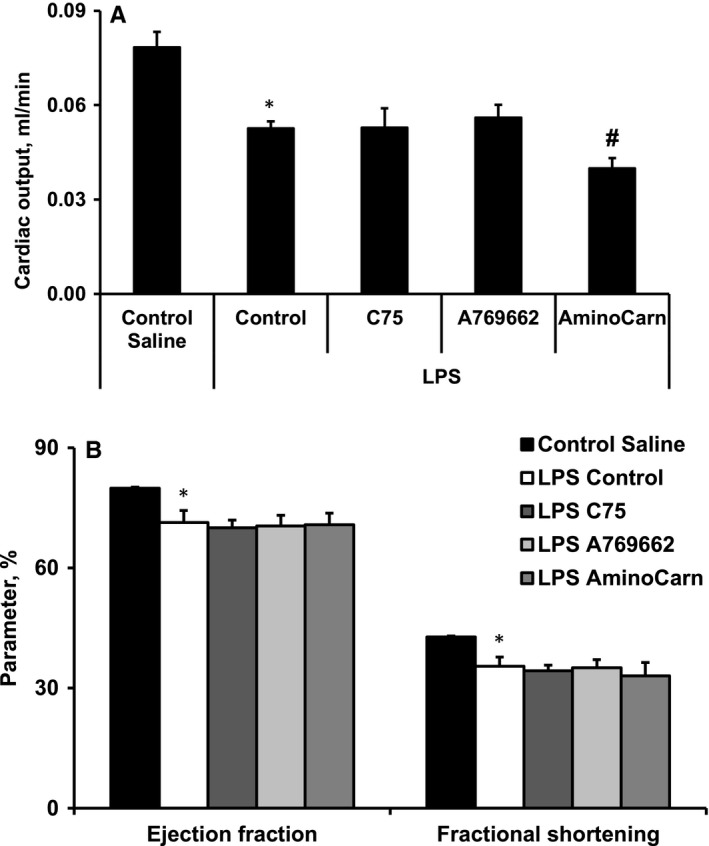
LPS‐induced decrease in cardiac output (A), ejection fraction and fractional shortening (B). Treatment with aminocarnitine worsened the cardiac output (A) without affecting the ejection fraction and fractional shortening (B). Each value represents the mean ± SEM of 5‐6 animals. *Significant difference between saline control and LPS control groups (Student's *t* test, *P* < .05). ^#^Significantly different from the LPS control group (ANOVA followed by Dunnett's test, *P* < .05)

The entire data set on echocardiographic measurements performed 4 hours after LPS administration is included in Table S1 in Appendix S1. Compared with saline control group, the significant decrease in end‐diastolic volume in the LPS‐treated group resulted in the reduced stroke volume, cardiac output, ejection fraction and fractional shortening by 36%, 33%, 11% and 17%, respectively (Figure 2, Table S1 in Appendix S1); meanwhile, the heart rate was significantly increased (Table S1 in Appendix S1). Compared with treatment with LPS, treatment with both C75 and A769662 did not affect the cardiac parameters (Figure 2, Table S1 in Appendix S1), while treatment with aminocarnitine significantly decreased heart rate and (Table S1 in Appendix S1), thus, worsened the cardiac output by an additional 24% (Figure 2A) without affecting the ejection fraction and fractional shortening (Figure 2B).

### 3.3 Fatty acid metabolism

**FIGURE 3:**
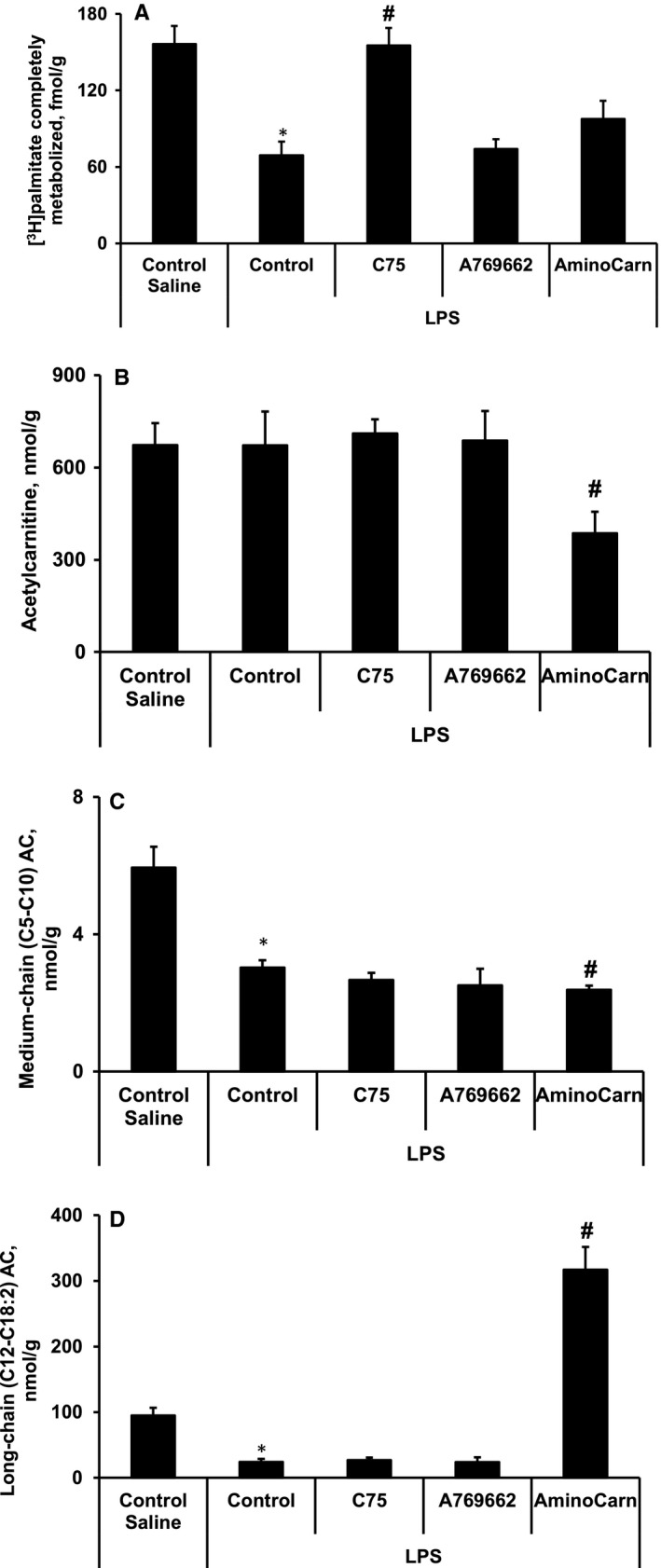
LPS‐induced inhibition of fatty acid metabolism in cardiac tissues. The [^3^H]‐palmitate oxidation in the heart in vivo (A) and the acylcarnitine profile (acetylcarnitine (B), medium‐ (C) and long‐chain (D) acylcarnitine) in the cardiac tissues. Treatment with C75 restored palmitate oxidation in the heart (A) without affecting the cardiac acylcarnitine profile (B‐D). The administration of aminocarnitine increased the content of long‐chain acylcarnitine (D) and decreased the medium‐chain acylcarnitine (C) and acetylcarnitine (B) contents, indicating the inhibition of CPT2‐dependent fatty acid metabolism. Each value represents the mean ± SEM of 5‐6 animals. *Significant difference between saline control and LPS control groups (Student's *t* test, *P* < .05). ^#^Significantly different from the LPS control group (ANOVA followed by Dunnett's test, *P* < .05)

To determine the compound treatment‐induced effects on fatty acid metabolism, we evaluated [^3^H]‐palmitate oxidation in the tissues in vivo and determined the acylcarnitine profile in the heart. As shown in Figure 3A, administration of LPS induced a decrease by 56% in cardiac palmitate oxidation compared to control group. The LPS‐induced inhibition of fatty acid metabolism was also confirmed by acylcarnitine profile measurements (Figure 3B‐D). The content of fatty acid metabolites, medium‐ and long‐chain acylcarnitine, was significantly reduced in the LPS‐treated hearts (Figure 3C,D), indicating the inhibition of CPT1‐dependent fatty acid metabolism.

Only treatment with C75 was able to restore palmitate oxidation in the heart (Figure 3A); however, compared to that of the LPS control group, no difference was observed in the cardiac acylcarnitine profile of the LPS + C75 group (Figure 3B‐D). In the aminocarnitine‐treated group, the content of long‐chain acylcarnitine was increased (Figure 3D), while the medium‐chain acylcarnitine and acetylcarnitine contents were decreased (Figure 3B,C), indicating the inhibition of CPT2‐dependent fatty acid metabolism in mitochondria.

### 3.4 Mitochondrial function

**FIGURE 4:**
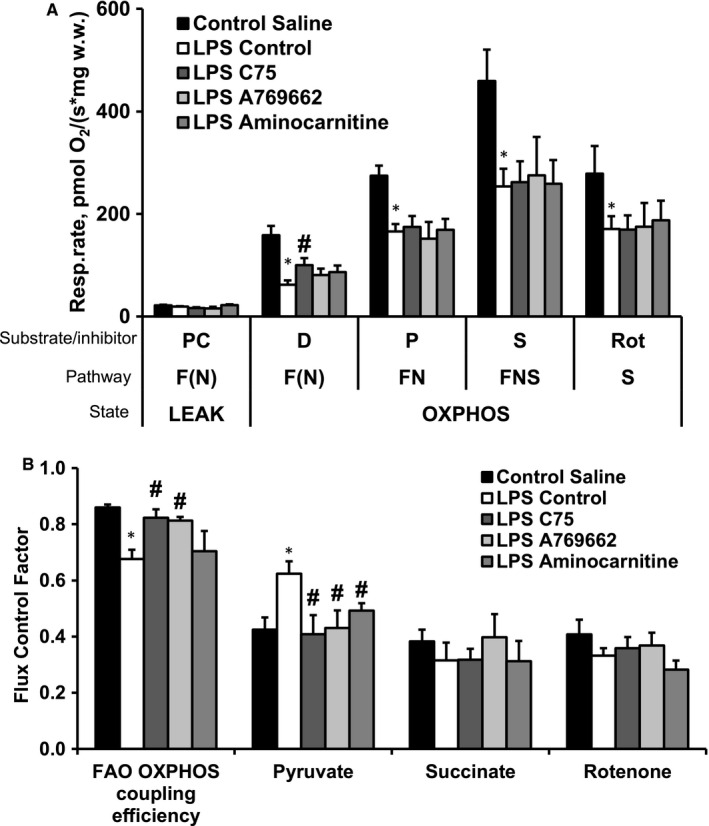
LPS‐induced changes in mitochondrial energy metabolism pattern in cardiac tissues. Respiration rate (A) and flux control factors (B) in permeabilized cardiac fibres. LPS administration decreased fatty acid oxidation pathway‐dependent oxidative phosphorylation (A,B) and induced pyruvate metabolism stimulation without affecting other pathways (B). Treatment with both C75 and A769662 restored the fatty acid oxidation pathway‐linked OXPHOS coupling efficiency and subsequently decreased pyruvate metabolism (B). Alongside after aminocarnitine administration, the fatty acid oxidation pathway‐linked OXPHOS coupling efficiency remained decreased, and pyruvate metabolism was inhibited (B). Each value represents the mean ± SEM of 5 animals. *Significant difference between saline control and LPS control groups (Student's *t* test, *P* < .05). ^#^Significantly different from the LPS control group (ANOVA followed by Dunnett's test, *P* < .05). FAO, fatty acid oxidation; F(N), fatty acid oxidation‐dependent pathway (FADH_2_ and NADH); N, NADH‐pathway; LEAK, substrate metabolism‐dependent state; OXPHOS, oxidative phosphorylation‐dependent state; D, ADP; P, pyruvate; PC, palmitoylcarnitine; Rot, rotenone; S, succinate

To further investigate the effects of the treatments on mitochondrial fatty acid oxidation, we assessed the mitochondrial function in permeabilized cardiac fibres. Consistent with the in vivo findings (Figure 3), FAO (F(N)) pathway‐dependent respiration (using palmitoylcarnitine as substrate) in the OXPHOS state was significantly decreased by 61% in the LPS control group (Figure 4A), which resulted in a 21% decrease in the FAO‐dependent OXPHOS coupling efficiency (Figure 4B) compared with the control group . Although the flux control factor analysis indicated that LPS administration induces pyruvate metabolism stimulation without affecting other pathways (Figure 4B), this increase was not sufficient to restore the LPS‐induced inhibition of FN and FNS pathway‐linked respiration in the OXPHOS state (Figure 4A). Although only treatment with C75 significantly improved the F(N) pathway‐linked respiration in the OXPHOS state (Figure 4A), treatment with both C75 and A769662 restored the F(N) pathway‐linked OXPHOS coupling efficiency and subsequently decreased pyruvate metabolism (Figure 4B). In addition, in the aminocarnitine‐treated group, the F(N) pathway‐linked OXPHOS coupling efficiency remained decreased, and pyruvate metabolism was inhibited, indicating the occurrence of energetic starvation in the heart.

## 4 DISCUSSION

The pathophysiology of sepsis/endotoxaemia‐induced cardiac dysfunction depends on the inflammatory response and disruption of mitochondrial fatty acid oxidation. However, although our study shows that FASN inhibition restores fatty acid metabolism in the heart, this is not sufficient to recover cardiac function under the conditions of endotoxaemia. In addition, we show that the accumulation of incomplete FA metabolism intermediates, namely long‐chain acylcarnitines, is more detrimental to cardiac function in endotoxaemia than decreased mitochondrial FAO alone.

Previous studies demonstrating inhibited fatty acid metabolism in hearts subjected to sepsis or endotoxaemia were mainly based on the analysis of protein/gene expression.^17^, ^32^, ^33^ Consistent with these findings, we demonstrate that LPS induces a functional impairment in FAO in the heart. In our study, the decreased respiration rate and OXPHOS coupling efficiency with palmitoylcarnitine indicate decreased mitochondrial FAO. Since palmitoylcarnitine is a substrate for CPT2, the endotoxaemia‐induced alterations in mitochondrial FAO are at the level and/or downstream of CPT2. However, the observed accumulation of long‐chain acylcarnitines after administration of CPT2 inhibitor to LPS‐treated animals indicates that FAO impairment in endotoxaemia is more likely downstream of CPT2. Nevertheless, the decreased content of long‐chain acylcarnitines in the heart also indicates reduced CPT1 activity in mice with endotoxaemia. Thus, stimulating the fatty acid metabolism pathway in mitochondria would be a strategy to restore overall ‘healthy’ cardiac FAO. In addition, it should be noted that although the observed inhibition of mitochondrial FAO is partially compensated by the stimulation of pyruvate metabolism, unfortunately, this is not sufficient to fully support the energy demand of normal cardiac function during endotoxaemia or sepsis.

The inhibition of CPT2‐dependent mitochondrial FAO by aminocarnitine exacerbates cardiac dysfunction. This phenomenon might be caused by two mechanisms. First, the inhibition of CPT2 induces an accumulation of long‐chain acylcarnitines, which have been shown to inhibit insulin signalling^34^ and mitochondrial pyruvate metabolism.^35^ In the present study, the inhibition of pyruvate metabolism induced by increased levels of long‐chain acylcarnitines results in an even more pronounced disturbances in energy metabolism in the aminocarnitine‐treated heart. Moreover, the inhibition of CPT2 might indirectly increase the expression of genes related to inflammation. Previously, it has been shown that long‐chain acylcarnitines activate proinflammatory signalling pathways in both immune and muscle cells.^36^, ^37^, ^38^ However, recent study in CPT2 KO C2C12 myotubes suggested that cell inflammation cannot be attributed to the accumulation of long‐chain acylcarnitines alone.^39^ Thus, the further up‐regulation of inflammation during endotoxaemia only partially could be related to the accumulation of long‐chain acylcarnitines in the heart. Interestingly, a higher concentration of acylcarnitines has been found in sepsis non‐survivors,^18^, ^40^ and taking into account that the concentration of circulating long‐chain acylcarnitines reflects their content in the heart,^30^ it could be expected that the accumulation of long‐chain acylcarnitines is detrimental to both cardiac function and sepsis/endotoxaemia outcomes.

Previously, strategies to inhibit FASN and activate AMPK have been demonstrated to have beneficial effects in experimental models of sepsis and endotoxaemia.^21^, ^26^, ^41^ In our study, none of the strategies was able to ameliorate LPS‐induced cardiac dysfunction, although, in the case of FASN inhibition, mitochondrial FAO and overall cardiac fatty acid metabolism were substantially improved. Obviously, the restoration of the FAO‐driven energy supply alone is not sufficient to recover cardiac mechanical function because it does not address the inflammation‐driven myocardial depression during sepsis/endotoxaemia. In contrast to previous studies,^21^, ^25^, ^26^ we did not observe any effect of compounds C75 and A769662 on the expression of inflammation‐related genes or on the levels of circulating inflammatory cytokines. These controversial results may be explained by the different models used, namely caecal ligation and puncture in previous studies vs LPS‐induced endotoxaemia in the current study. Thus, the lack of anti‐inflammatory effects in our study might explain why no effects on cardiac function were observed. Overall, these data show that for the recovery of cardiac function, the combination of restoring energy metabolism and anti‐inflammatory effects is necessary.

In conclusion, the present study demonstrates that the inhibition of mitochondrial FAO, in particular CPT2‐dependent FAO, is detrimental to cardiac function in endotoxaemia and sepsis. Thus, the stimulation of FAO is essential to ameliorate the disturbances in energy metabolism in the inflamed heart; however, it is not sufficient to rescue endotoxaemia‐induced cardiac dysfunction. Thus, additional therapy, probably anti‐inflammatory therapy, might be required to ameliorate cardiac dysfunction and reduce organ injury in patients with endotoxaemia and sepsis.

## CONFLICT OF INTEREST

The authors confirm that there are no conflicts of interest.

## AUTHOR CONTRIBUTIONS

**Marina Makrecka‐Kuka:** Conceptualization (lead); Data curation (lead); Formal analysis (lead); Funding acquisition (lead); Investigation (lead); Methodology (lead); Project administration (lead); Resources (equal); Supervision (lead); Validation (lead); Visualization (lead); Writing‐original draft (lead); Writing‐review & editing (equal). **Stanislava Korzh:** Formal analysis (equal); Investigation (equal); Validation (equal); Visualization (equal); Writing‐review & editing (equal). **Melita Videja:** Formal analysis (equal); Investigation (equal); Validation (equal); Writing‐review & editing (equal). **Reinis Vilskersts:** Data curation (equal); Formal analysis (equal); Investigation (equal); Methodology (equal); Validation (equal); Writing‐review & editing (equal). **Eduards Sevostjanovs:** Data curation (equal); Formal analysis (equal); Investigation (equal); Methodology (equal); Validation (equal); Writing‐review & editing (equal). **Olga Zharkova‐Malkova:** Data curation (equal); Investigation (equal). **Pavel Arsenyan:** Methodology (equal); Resources (equal); Writing‐review & editing (equal). **Janis Kuka:** Conceptualization (supporting); Validation (equal); Writing‐review & editing (equal). **Maija Dambrova:** Conceptualization (supporting); Methodology (supporting); Supervision (equal); Writing‐review & editing (equal). **Edgars Liepinsh:** Conceptualization (supporting); Methodology (supporting); Project administration (supporting); Supervision (equal); Validation (equal); Writing‐review & editing (equal).

## Supporting information

Appendix S1

## Data Availability

The data sets generated and analysed during the current study are available from the corresponding author on reasonable request.
