# Supplementary material for: Inhibition of CPT2 exacerbates cardiac dysfunction and inflammation in experimental endotoxaemia
Source: J Cell Mol Med. 2020 Sep 7;24(20):11903–11. doi: 10.1111/jcmm.15809 (PMC7578905; doi:10.1111/jcmm.15809)
Supplement: Supplementary file 1 — Appendix S1 [file JCMM-24-11903-s001.pdf]

Inhibition of CPT2 exacerbates cardiac dysfunction and inflammation in experimental sepsis

Marina Makrecka-Kuka[1,*], Stanislava Korzh[1], Melita Videja[1,2], Reinis Vilskersts[1,2], Eduards Sevostjanovs[1], Olga Zharkova-Malkova[1], Pavel Arsenyan[1], Janis Kuka[1], Maija Dambrova[1,2], Edgars Liepinsh[1]

[1] Latvian Institute of Organic Synthesis, Aizkraukles Str. 21, Riga, LV-1006, Latvia
[2] Riga Stradins University, Faculty of Pharmacy, Dzirciema Str 16, Riga, LV-1007, Latvia

*Corresponding author: Marina Makrecka-Kuka, Ph.D.
Latvian Institute of Organic Synthesis,
Aizkraukles Str. 21, Riga LV-1006, Latvia
Phone/fax: +371 66155159
E-mail address: makrecka@farm.osi.lv

Supporting Information

Methods

Synthesis of aminocarnitine

Straightforward, totally stereo controlled synthesis of (*R*)-aminocarnitine was performed in 5 sequential steps from carnitine (Castagnani et al., 1995). Semi pure product was isolated as brownish foam. Taking in mind a necessity to utilize as pure as possible material in pharmacological studies this technical (*R*)-aminocarnitine was modified to Cbz-protected derivative, purified by reverse-phase chromatography on C18-silica gel to reed off all impurities. Then Cbz-protecting group was removed by bubbling of hydrogen gas in methanol solution of Cbz-(*R*)-aminocarnitine in the presence of palladium/charcoal (10%, wet). Finally, pure (*R*)-aminocarnitine was obtained as colourless foam after simple filtration, stirring of solution on Si-TMT (transition metal scavenger) and evaporation of a solvent.

Castagnani, R., et al., *Stereospecific synthesis of (R)-aminocarnitine (emeriamine) starting from (R)-carnitine via double inversion of configuration.* Journal of Organic Chemistry, **1995**. 60(25): 8318-8319.

Gene accession number and primer sequences of qPCR primers

Gene symbol	Full name	NCBI Accession number	Forward primer sequence (5'->3')	Reverse primer sequence (5'->3')	Amplicon length, b
β-actin	Beta actin	NM_007393.5	CCTCTATGCCAACACAGTGC	CATCGTACTCCTGCTTGCTG	215
TNFα	Tumor necrosis factor	NM_013693.3	GACCCTCACACTCAGATCATCTTCT	CCTCCACTTGGTGGTTTGCT	80
IL6	Interleukin-6	NM_001314054.1	TCTATACCACTTCACAAGTCGGA	GAATTGCCATTGCACAACTCTTT	88
Il1β	Interleukin-1-beta	NM_008361.4	GGGCCTCAAAGGAAAGAATC	TTGCTTGGGATCCACACTCT	88

Ye J, et al., *Primer-BLAST: a tool to design target-specific primers for polymerase chain reaction.* BMC Bioinformatics. **2012**;13(1):134.

doi:10.1186/1471-2105-13-134

Results

Supplementary Table S1. Echocardiographic heart parameters 4 h after LPS administration

	**Control**	**LPS 10 mg/kg**			
	**Saline**	**Control**	**C75**	**A769662**	**AminoCarnitine**
Heart rate, bpm	473±17	516±11*	517±20	504±21	420±13#
LVPWs, mm	1.40±0.09	1.08±0.07*	1.15±0.07	1.16±0.07	1.20±0.07
LVPWd, mm	0.69±0.05	0.67±0.03	0.72±0.05	0.69±0.06	0.82±0.04#
LVIDs, mm	2.51±0.03	2.49±0.12	2.52±0.05	2.59±0.13	2.44±0.15
LVIDd, mm	4.38±0.06	3.85±0.07*	3.84±0.08	3.98±0.11	3.76±0.12
IVSs, mm	1.37±0.10	1.31±0.06	1.30±0.03	1.29±0.03	1.33±0.05
IVSd, mm	0.67±0.03	0.72±0.02	0.75±0.03	0.71±0.03	0.80±0.05
ESV, ml	0.042±0.002	0.042±0.006	0.043±0.002	0.047±0.007	0.041±0.007
EDV, ml	0.200±0.014	0.144±0.008*	0.144±0.009	0.159±0.012	0.136±0.013
Stroke volume, ml	0.158±0.013	0.102±0.005*	0.101±0.008	0.112±0.008	0.096±0.007

Left ventricular posterior wall thickness at end-systole (LVPWs) and at end-diastole (LVPWd), left ventricular internal dimension at end-systole (LVIDs) and at end-diastole (LVIDd), interventricular septal thickness at end-systole (IVSs) and at end-diastole (IVSd), left ventricular volume at end-systole (ESV) and at end-diastole (EDV), and stroke volume calculated as difference between EDV and ESV 4 h after LPS administration. Each value represents the mean ± SEM of 5-6 animals. *Significant difference between saline control and LPS control groups (Student's t-test, P<0.05); #Significantly different from the LPS control group (ANOVA followed by Dunnett's test, P < 0.05).
